# Impact of the COVID‐19 Pandemic on Conduct and Results of CLEAR Outcomes Trial

**DOI:** 10.1002/clc.24328

**Published:** 2024-07-30

**Authors:** Abhayjit Singh, Luke J. Laffin, Ashish Sarraju, A. Michael Lincoff, Stephen J. Nicholls, LeAnne Bloedon, William J. Sasiela, Na Li, Paula Robinson, Stephanie Kelly, Denise Mason, Steven E. Nissen

**Affiliations:** ^1^ Section of Preventive Cardiology and Rehabilitation, Department of Cardiovascular Medicine Cleveland Clinic Foundation Cleveland Ohio USA; ^2^ Cleveland Clinic Coordinating Center for Clinical Research (C5Research) Cleveland Ohio USA; ^3^ Victorian Heart Institute Monash University Melbourne Victoria Australia; ^4^ Esperion Therapeutics Inc. Ann Arbor Michigan USA

**Keywords:** bempedoic acid, cardiovascular outcomes, clinical trial, COVID‐19, hyperlipidemia, statin intolerance

## Abstract

**Introduction:**

The COVID‐19 pandemic disrupted clinical research. CLEAR Outcomes investigated the effect of bempedoic acid (BA) versus placebo in 13 970 patients with statin intolerance and high cardiovascular (CV) risk. BA reduced the risk of the primary endpoint (composite of CV death, nonfatal myocardial infarction, nonfatal stroke, or coronary revascularization) by 13%. CLEAR Outcomes began before and continued for 2.7 years after the start of the pandemic.

**Methods:**

The impact of the COVID‐19 pandemic on patient disposition, adverse events, and major adverse CV events (MACE) in CLEAR Outcomes was assessed.

**Results:**

Rates of severe infection, hospitalization, or first MACE associated with a positive COVID‐19 test were low and balanced between treatment groups. Rates of all‐cause death, non‐CV death, and undetermined death increased in the pandemic period compared with the pre‐pandemic period, while rates of CV death with a known etiology remained stable. A sensitivity analysis excluding undetermined deaths occurring after the onset of the pandemic from the CV death designation yielded hazard ratios of 0.84 (95% CI, 0.76–0.93) for the primary endpoint and 0.94 (95% CI, 0.76–1.16) for the secondary endpoint of CV death, compared with 0.87 (95% CI, 0.79–0.96) and 1.04 (95% CI, 0.88–1.24), respectively, in the original analysis.

**Conclusion:**

The CLEAR Outcomes trial continued uninterrupted throughout the COVID‐19 pandemic. Certain trial endpoints may have been impacted by the pandemic. Specifically, the classification of undetermined deaths as CV deaths may have attenuated the effect of BA on key efficacy endpoints.

## Graphical abstract

The CLEAR Outcomes trial was conducted uninterrupted during the COVID‐19 pandemic due to key changes in study procedures. The definitions of the trial endpoints were not altered for the pandemic, raising the possibility that outcomes related to cardiovascular mortality may have been impacted.

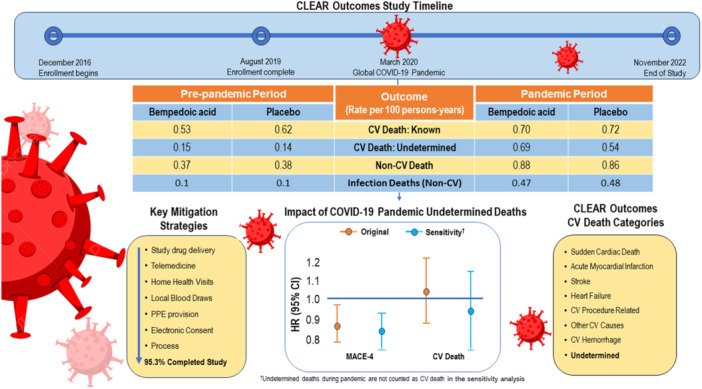

## 1 Introduction

COVID‐19, a respiratory illness caused by the SARS‐CoV‐2 virus, was first identified in December 2019 and spread rapidly across the globe. A pandemic was declared by the World Health Organization (WHO) in March 2020 and impacted most aspects of healthcare, from office visits to elective procedures to clinical research. The combination of fear surrounding a novel disease, governmental restrictions in place to reduce virus transmission, and reallocation of resources to combat the pandemic resulted in significant challenges in the enrollment and conduct of clinical trials [1]. Pandemic‐related isolation restrictions further complicated trial conduct by posing challenges in ensuring patients received study drug and procedures as planned and likely affected the accurate ascertainment of clinical outcomes and endpoints.

**Figure 1:**
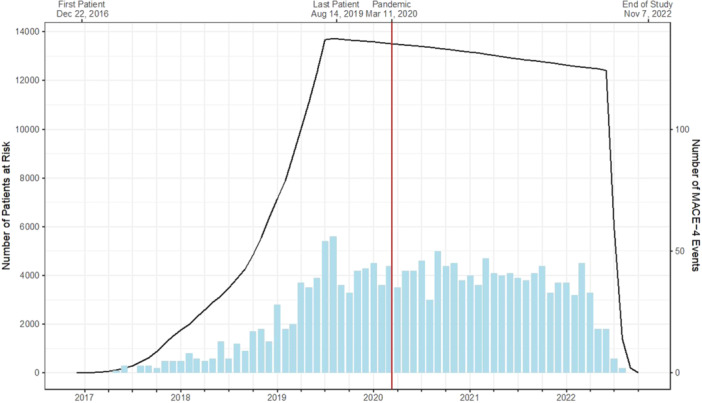
CLEAR Outcomes participant enrollment and follow‐up. The CLEAR Outcomes trial began enrollment in December 2016 and completed follow‐up in November 2022. The line graph displays the cumulative number of participants at risk for the primary outcome, which was a composite of the following major adverse cardiovascular events (MACEs): cardiovascular death, myocardial infarction, stroke, or coronary revascularization. The bar graph displays the number of MACEs per time period (1 month).

The CLEAR (Cholesterol Lowering via Bempedoic Acid (ECT1002), an ACL–Inhibiting Regimen) Outcomes trial assessed the impact of bempedoic acid, among high cardiovascular (CV) risk patients with statin intolerance, on the incidence of a four‐component primary endpoint of major adverse CV events (MACE‐4), defined as death from CV causes, nonfatal myocardial infarction, nonfatal stroke, or coronary revascularization [2]. CLEAR Outcomes enrolled 13 970 participants at 1250 sites in 32 countries from December 2016 until August 2019, with trial completion occurring in November 2022. A substantial proportion of trial follow‐up (and MACE accrual) occurred during the COVID‐19 pandemic amidst the associated isolation restrictions worldwide (Figure 1).

We sought to assess the impact of COVID‐19 infections, along with the pandemic itself, on the CLEAR Outcomes trial. This included assessing the number of patients who discontinued the study drug or trial participation, experienced a first MACE near the time of COVID infection, or suffered an adverse event attributable to COVID‐19. Further, the increase in worldwide mortality seen during the pandemic appeared to be greater than the deaths due to confirmed COVID‐19 infections [3]. We assessed whether this excess death rate, particularly the number of deaths with an undetermined etiology, impacted the clinical endpoint of CV death in CLEAR Outcomes.

## 2 Methods

The COVID‐19 pandemic prompted the CLEAR Outcomes team to implement mitigation strategies supporting participant safety, protocol adherence, and patient retention. These strategies included decentralized services such as delivery of study drugs directly to patients, allowing local blood draws, enabling use of home health visits, use of telemedicine, obtaining consent electronically, and providing patients with personal protective equipment. CLEAR Outcomes was approved by the ethics committees at the participating sites, and all trial participants provided informed consent to participate in the trial. To assess the impact of the COVID‐19 pandemic on the conduct of CLEAR Outcomes, the full analysis and safety analysis data sets were reviewed. The number of participants who either discontinued the assigned treatment or ceased participation in the clinical trial altogether due to the pandemic was tabulated. Trial safety data assessed the number of patients, as determined by trial investigators, who suffered an adverse event related to COVID‐19. COVID‐19 adverse events were based on the high‐level term of coronavirus infection using Medical Dictionary for Regulatory Activities (MedDRA) version 23.1. COVID‐19 infection was confirmed if a participant reported a positive polymerase chain reaction (PCR) test result.

During CLEAR Outcomes, all study deaths were adjudicated by a clinical events committee (CEC) to determine if the death was CV or non‐CV in nature. In instances when there was very limited or no information available regarding the cause of death, the death was classified as an undetermined cause of death. Consistent with other contemporary cardiometabolic clinical trials, such deaths were categorized as a CV death per the CEC charter. Following the onset of the pandemic, the CEC issued guidance to help properly adjudicate deaths due to COVID‐19 infection when information was available. To determine the pandemic's impact on the rates of accrued clinical endpoints, three different time periods were considered. These consisted of a pre‐pandemic period (before March 11, 2020, the date the WHO declared COVID‐19 a global pandemic) and two approximately equal pandemic periods based on the remaining total trial follow‐up (March 11, 2020 to June 30, 2021, and July 1, 2021 to trial completion). Death rates were calculated for CV and non‐CV causes, expressed as number of events per 100 patient‐years. To further evaluate the impact of the high proportion of deaths of undetermined etiology, a sensitivity analysis was performed, which did not count undetermined deaths in the pandemic period as CV deaths.

## 3 Results

Of the 13 970 participants enrolled in CLEAR Outcomes, 95.3% of participants completed the study and 69.6% of participants completed study treatment. Vital status was available for 13 886 participants (99.4%) at trial completion. Of the 4246 participants who discontinued treatment with the study drug (2037 assigned to bempedoic acid group and 2209 assigned to placebo), 274 patients (2% of all enrolled participants) indicated that the COVID‐19 pandemic was their primary reason for drug discontinuation. Of the 653 participants who did not complete the study, 16 patients (0.1% of those enrolled) cited the pandemic as the primary reason for study withdrawal (Table 1).

**Table 1:** Impact of COVID‐19 pandemic on patient disposition, major adverse cardiovascular events, and adverse events.

	Bempedoic acid	Placebo	Total participants
*Full analysis set—randomized participants, no*.	6992	6978	13 970
Participants who did not complete study drug treatment, no. (%)	2037 (29.1)	2209 (31.7)	4246 (30.4)
Reported pandemic as primary reason, no. (%)	151 (2.2)	123 (1.8)	274 (2.0)
Participants who did not complete the trial, no. (%)	295 (4.2)	358 (5.1)	653 (4.7)
Reported pandemic as primary reason, no. (%)	7 (0.1)	9 (0.1)	16 (0.1)
Participants with MACE‐4 near the time of confirmed COVID‐19 infection,^a^, ^b^ no. (%)	10 (0.1%)	11 (0.2%)	21 (0.2%)
COVID‐19 as reported event leading to death, no. (%)	60 (0.9)	58 (0.8)	118 (0.8)
*Safety analysis set—treated participants, no*.	7001	6964	13 965
Participants with reported adverse event of COVID‐19, no. (%)	790 (11.3)	876 (12.6)	1666 (11.9)
Confirmed^a^ COVID‐19 infection, no. (%)	543 (7.8)	606 (8.7)	1149 (8.2)
Serious COVID‐19 adverse event, no. (%)	151 (2.2)	175 (2.5)	326 (2.3)
Hospitalized with confirmed COVID‐19 infection, no. (%)	87 (1.2)	95 (1.4)	182 (1.3)

^a^ Confirmed COVID‐19 infection as determined by participant reporting of COVID‐19 as an adverse event and a positive result on PCR test for COVID‐19 infection. The timing (proximity) of the COVID‐19 PCR result and AE were not evaluated.

^b^ Between 14 days before and 30 days following confirmed COVID‐19 infection.

COVID‐19 infection was the most frequent adverse event observed in CLEAR Outcomes, affecting approximately 12% of all participants (11.3% and 12.6% in the bempedoic acid and placebo groups, respectively). Of these, 2.3% met the criteria as a serious adverse event. As shown in Table 1, the rates of serious COVID‐19 infection, study drug discontinuation, and trial withdrawal due to COVID‐19 were similar in both the bempedoic acid and placebo study arms. Among the 1149 patients with PCR–positive COVID‐19 infection, 64 patients (5.5%, 30 bempedoic acid–assigned patients and 34 placebo‐assigned patients) were adjudicated to have any MACE in the 6‐week peri‐infection period (14 days before positive PCR test and up to 30 days following). Similarly, of the 182 trial participants hospitalized with a confirmed COVID‐19 infection, five patients (two receiving bempedoic acid and three receiving placebo) were documented as having any MACE during the hospitalization. Regional rates of PCR–positive COVID‐19 infection were highest in Western Europe (13.1% of its enrolled participants) and lower in Central and Eastern Europe (9.5%), Latin America (6.0%), and North America (5.3%).

Ultimately, 858 participant deaths were reviewed by the CEC, including 436 deaths in the bempedoic acid cohort and 422 deaths in the placebo cohort. Of the 858 deaths, 530 were classified as CV deaths. CV death was subdivided per the CEC Charter into eight categories including sudden cardiac death, acute myocardial infarction, stroke, heart failure, CV procedure, other CV causes, CV hemorrhage, and undetermined. The most common category in each treatment group was “undetermined” (212 deaths [24.7% of total] with 118 of 271 CV deaths in the bempedoic acid group and 94 of 259 CV deaths in the placebo group). Stratified pre‐pandemic and pandemic period death rates are displayed in Table 2. The rate of total deaths increased in both pandemic periods compared to the pre‐pandemic period. All‐cause death rates increased from 1.04 (bempedoic acid) and 1.14 (placebo) per 100 person‐years during the pre‐pandemic period (before March 11, 2020) to 2.30 (bempedoic acid) and 1.95 (placebo) during the first pandemic period (March 11, 2020 to June 30, 2021) and 2.23 (bempedoic acid) and 2.38 (placebo) during the second pandemic period (after June 30th, 2021). With respect to death from non‐CV causes, the rate of death from infectious or pulmonary causes increased substantially from the pre‐pandemic to pandemic periods, while the rate of death from all other non‐CV causes increased only slightly during the pandemic.

**Table 2:** Risk of death from various causes by pandemic period.

		Number of events (rate per 100 person‐years)					
Period	Treatment group	All‐cause death	Undetermined cause of death^a^	CV death with known cause	Non‐CV death	Non‐CV death: Infection and pulmonary causes	Non‐CV death: All other causes
Overall trial	Bempedoic acid (23 944 patient‐years)	1.81	0.49	0.63	0.69	0.34	0.35
	Placebo (23 833 patient‐years)	1.76	0.39	0.69	0.68	0.34	0.34
Pre‐pandemic (before March 11, 2020)	Bempedoic acid (8919 patient‐years)	1.04	0.15	0.53	0.37	0.10	0.27
	Placebo (8886 patient‐years)	1.14	0.14	0.62	0.38	0.10	0.28
Pandemic period 1 (March 11, 2020 to June 30, 2021)	Bempedoic acid (8745 patient‐years)	2.30	0.59	0.75	0.95	0.58	0.37
	Placebo (8730 patient‐years)	1.95	0.44	0.74	0.77	0.50	0.26
Pandemic period 2 (after July 1, 2021)	Bempedoic Acid (6281 patient‐years)	2.23	0.83	0.62	0.78	0.33	0.45
	Placebo (6267 patient‐years)	2.38	0.69	0.70	0.99	0.45	0.54

Abbreviation: CV, cardiovascular.

^a^ Undetermined cause of death was assigned as cardiovascular death according to the Clinical Events Committee Charter.

**Figure 2:**
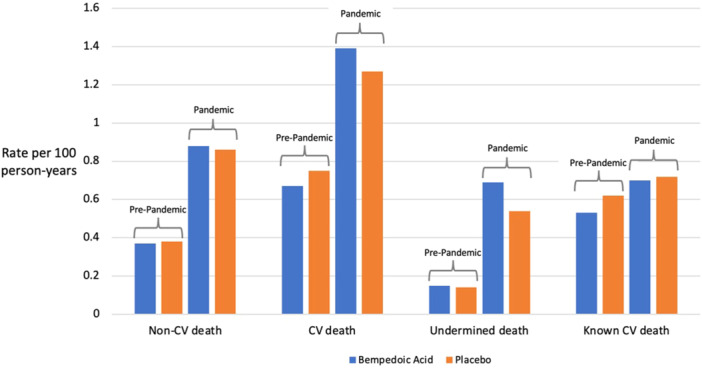
Event rates among CLEAR Outcomes participants. Death rates among trial participants randomized to placebo or bempedoic acid are shown in the pre‐pandemic and combined pandemic time periods. Death rates are categorized into non‐cardiovascular death and cardiovascular death, and cardiovascular deaths are further categorized as known CV cause or undetermined.

The rate of CV death also increased following the onset of the COVID‐19 pandemic. However, when separated into deaths with a known CV cause and deaths with an undetermined cause (but per CEC charter classified under CV death), the rate of undetermined deaths increased significantly during the pandemic (Figure 2) while the rate of known CV death remained relatively unchanged.

Prior to the declaration of the pandemic, the rate of undetermined death was low in both groups (0.15 in patients receiving bempedoic acid and 0.14 in patients receiving placebo per 100 persons‐years). Following the onset of the pandemic, this rate increased substantially to 0.69 in the bempedoic acid cohort and 0.54 in the placebo cohort (weighted average of first and second pandemic periods). Deaths attributed to infection increased from a rate of 0.1 per 100 persons‐years pre‐pandemic in both treatment groups to 0.47 in the bempedoic acid group and 0.48 in the placebo group during the pandemic. In contrast, the rate of death from known CV causes remained relatively stable at 0.53 and 0.62 in the pre‐pandemic period and at 0.70 and 0.72 in the pandemic period for the bempedoic acid and placebo groups, respectively. Regional rates of undetermined deaths, as a percentage of total CV deaths, were similar among North America (40%), Latin America (35%), and Central and Eastern Europe (45%), but notably lower among Western Europe trial participants (19%).

To account for the observed increase in deaths of undetermined etiology driven by the pandemic, a sensitivity analysis was performed, which excluded undetermined deaths that occurred during this period. Because CV death was included in both the primary composite endpoint (MACE‐4) and the first key secondary endpoint (MACE‐3; death from CV cause, nonfatal stroke, or nonfatal myocardial infarction) in the original statistical analysis plan, these endpoints were reassessed as part of the sensitivity analysis (Table 3). Removing undetermined deaths after the start of the pandemic resulted in hazard ratios (HRs) of 0.94 (95% confidence interval [CI], 0.76–1.16; *p* = 0.37) for CV death, 0.84 (95% CI, 0.76–0.93; *p* = 0.0005) for MACE‐4, and 0.81 (95% CI, 0.72–0.91; *p* = 0.0006) for MACE‐3.

**Table 3:** Original and sensitivity analyses of bempedoic acid treatment on primary endpoint (MACE‐4), key secondary endpoint (MACE‐3), and cardiovascular death.

Clinical endpoint (original analysis)	Bempedoic acid (*N* = 6992)	Placebo (*N* = 6978)	Hazard ratio (95% CI)
Primary endpoint (MACE‐4), no. (%)	819 (11.7)	927 (13.3)	0.87 (0.79, 0.96)
Key secondary endpoint (MACE‐3), no. (%)	575 (8.2)	663 (9.5)	0.85 (0.76, 0.96)
Cardiovascular death, no. (%)	269 (3.8)	257 (3.7)	1.04 (0.88, 1.24)
Clinical endpoint (sensitivity analysis, which excludes undetermined deaths during the pandemic)			
Primary endpoint (MACE‐4), no. (%)	725 (10.4)	852 (12.2)	0.84 (0.76, 0.93)
Key secondary endpoint (MACE‐3), no. (%)	481 (6.9)	586 (8.4)	0.81 (0.72, 0.91)
Cardiovascular death, no. (%)	165 (2.4)	176 (2.5)	0.94 (0.76, 1.16)

*Note:* MACE‐4 includes death from cardiovascular causes, nonfatal myocardial infarction, nonfatal stroke, or coronary revascularization. MACE‐3 includes death from cardiovascular causes, nonfatal myocardial infarction, and nonfatal stroke.

Abbreviation: MACE, major adverse cardiovascular events.

## 4 Discussion

Cardiovascular outcomes trials of novel therapeutics enroll large numbers of participants who are followed for long periods of time. Unlike some contemporaneous clinical trials, CLEAR Outcomes was not suspended during the COVID‐19 pandemic and achieved high levels of patient follow‐up and trial completion rates [4]. More than 2.5 years of the trial follow‐up period coincided with the pandemic, and more than 10% of study participants reported contracting a COVID‐19 infection during that time. The low rate of pandemic‐related study drug discontinuations and trial withdrawals in CLEAR Outcomes was due in large part to mitigation strategies designed to support participant safety, protocol adherence, and patient retention. As CLEAR Outcomes was a multinational trial, remediations were implemented in accordance with global and local laws and regulations, as well as in alignment with global regulatory guidance issued by the US Food and Drug Administration [5] and the European Medicines Agency [6]. The collaborative efforts of the clinical study sites, regulatory authorities, vendors, Cleveland Clinic Coordinating Center for Clinical Research (C5R), and the trial sponsor (Esperion Therapeutics) allowed for the swift implementation of mitigation strategies. Services that were implemented included electronic consent, study drug delivery, home health visits, and telemedicine, which collectively ensured patient safety and data integrity. These measures also enabled sites to stay better connected with, and accessible to, their study participants. While CLEAR Outcomes was not immune to the effects of the pandemic, the success of these mitigation strategies may provide valuable insights for maintaining resiliency of clinical research during future public health emergencies.

A retrospective analysis of 62 252 clinical trial activations found that during the initial months of the COVID‐19 pandemic (February 2020 through May 2020), trial activations for US–based studies were only 57% of the expected estimate when compared with pre‐pandemic activations [4]. Another study of 321 218 non‐COVID‐19 clinical trials found that among trials stopped from January 2017 to May 2020, an average of 1147 trials per month were stopped during the start of the pandemic (the first 5 months of 2020), compared with 638 trials per month from 2017 to 2019 [7]. A more recent analysis sought to quantify the impact of the pandemic on industry‐sponsored clinical trials, demonstrating that year‐over‐year clinical trial screening rates declined sharply from 2019 to 2020, with a slight rebound in 2021 [8].

Considering CLEAR Outcomes continued for 2.7 years after the onset of the COVID‐19 pandemic, when significant accrual of MACE events occurred, it is appropriate to assess any potential impact of this unprecedented event on primary and key secondary endpoints. Based on the pre‐specified statistical analysis plan described in the primary manuscript, treatment with bempedoic acid did not affect the risk of CV death (a secondary endpoint) compared with placebo. Two hundred and sixty‐nine CV deaths occurred in participants assigned bempedoic acid (3.8%) versus 257 CV deaths among participants assigned placebo (3.7%) (HR 1.04; 95% CI, 0.88–1.24) [2]. The lack of an isolated CV mortality benefit is consistent with the findings of other contemporary CV outcomes trials, including the IMPROVE‐IT [9], FOURIER [10], and ODYSSEY Outcomes trials [11].

The rate of all‐cause mortality during CLEAR Outcomes was approximately twice as high following the onset of the pandemic compared to the pre‐pandemic period. Similar or even greater increases were noted in the pandemic death rates attributable to pulmonary/infectious causes and undetermined causes (which were classified as CV deaths). Meanwhile, the rate of CV deaths due to a known cause remained relatively stable throughout the pre‐pandemic and pandemic periods, with similar rates in both treatment arms. In CLEAR Outcomes, the rate of undetermined cause of death represented 24.7% of all trial deaths. This percentage is significantly larger than in other trials, demonstrated by a pooled analysis of nine global clinical cardiometabolic trials performed pre‐pandemic (between 2009 and 2017), which found that 15.6% of deaths were attributable to undetermined causes (ranging between 7% and 22%) [12]. Pandemic‐associated disruptions were likely responsible for the high rate of undetermined deaths in CLEAR Outcomes. Data from the early stages of the pandemic suggest isolation precautions and pandemic‐related restrictions resulted in more patients dying at home compared with prior years [13]. Other data demonstrate decreases in healthcare utilization during the COVID‐19 pandemic, resulting in less patient contact with the health system. These issues, together with other challenges imposed by the COVID‐19 pandemic, likely complicated the precise adjudication of many trial deaths.

The increase in death rates observed during the pandemic period of the CLEAR Outcomes trial mirrors the excess mortality seen globally during the COVID‐19 pandemic. During the years 2020 and 2021, the WHO estimated a total of 14.83 million excess deaths associated directly or indirectly with the COVID‐19 pandemic, a number nearly threefold greater than the 5.42 million deaths directly attributed to COVID‐19 infection for the same period [14]. Additionally, the mean age of participants enrolled in CLEAR Outcomes was 65.5 years, rendering them vulnerable to an increased risk of death from COVID‐19, as retrospective analyses have demonstrated that patients in this age range had a higher incidence of excess death compared with younger patients during the pandemic [15]. Of note, the true number of COVID infections in CLEAR Outcomes likely exceeded those confirmed with PCR testing, particularly early in the pandemic when test availability was limited. The above issues may have complicated the precise determination of the cause of death in many cases. As the etiology of mortality is often a crucial clinical endpoint in trials, even small inaccuracies may significantly impact study conclusions.

Overall, these findings suggest that many of the undetermined deaths (that are classified as CV deaths) occurring after the onset of the pandemic may represent COVID‐19 infection or pandemic‐related fatalities. Although the exact etiology of these deaths will remain uncertain, the similar pre‐pandemic and pandemic death rates from known CV causes, together with the finding that confirmed COVID‐19 infections (and hospitalizations) did not precipitate a meaningful number of peri‐infection events, suggests that these undetermined deaths may not represent a CV death when considered in the context of the pandemic.

Regional differences observed in Western Europe may also reflect the above phenomenon. When assessed by region, PCR–positive COVID‐19 infections were highest in areas where the percentage of unknown CV deaths was lowest. This suggests that a higher rate of accurately diagnosing COVID‐19 may lead to less participants with an unknown cause of death.

The higher rate of undetermined death in participants receiving bempedoic acid (0.69 per 100 person‐years, versus 0.54 in participants receiving placebo) in the pandemic period was likely due to chance. Rates of both overall and serious adverse events of COVID‐19 infection were similar among treatment groups, as was the rate of non‐CV death overall. Moreover, there is no biological plausibility or observational data to suggest that treatment with bempedoic acid would place patients at higher risk of complications from COVID‐19 or other infections. Rather, retrospective observational data found that treatment with statins, which are lipid‐lowering therapies similar to bempedoic acid, was associated with decreased mortality in hospitalized COVID‐19 patients [16].

Finally, categorizing undetermined deaths as CV may have influenced the observed effect of bempedoic acid treatment on the endpoint of CV death (and thus the associated primary MACE‐4 composite endpoint and the key secondary MACE‐3 endpoint). It has been previously suggested that the inclusion of undetermined deaths as CV deaths may result in a bias toward the null [17]. Considering the high rate of undetermined deaths in CLEAR Outcomes, this issue may have been further exaggerated by the COVID‐19 pandemic.

The sensitivity analysis performed to exclude all undetermined deaths (in both treatment groups) occurring during the pandemic period further supports this notion, finding that treatment with bempedoic acid resulted in hazard ratios of 0.94 for CV death (*p* = 0.37), 0.84 (*p* = 0.0005) for MACE‐4 and 0.81 (*p* = 0.0006) for MACE‐3 endpoints. This sensitivity analysis raises the question as to whether treatment with bempedoic acid in CLEAR Outcomes may have had a greater impact on CV death and composite CV events if not for the COVID‐19 pandemic. It is not yet known whether this observation holds true in other contemporary CV outcome trials that significantly overlapped with the pandemic. Further research is needed to fully understand the impact of the pandemic on clinical trial outcomes.

## 5 Conclusion

The COVID‐19 pandemic disrupted nearly every aspect of clinical care and research across the globe. The lasting impact of the pandemic on the clinical trial landscape will likely remain unknown for years. Despite the mitigations put in place to minimize disruptions in the CLEAR Outcomes trial, the excess mortality documented during the pandemic, along with pandemic‐associated isolation restrictions, likely diluted the effect of bempedoic acid treatment on the endpoint of CV death and associated composite endpoints in this trial. As more data emerge, medical and research communities will hopefully be better equipped to manage and mitigate any clinical trial–related risks and challenges in outcome assessment associated with future large‐scale public health emergencies.

## Conflicts of Interest

Luke J. Laffin served as a consultant and/or served on steering committees for Medtronic, Lilly, Mineralys Therapeutics, AstraZeneca, Idorsia, and Crispr Therapeutics; received research funding from AstraZeneca; and has ownership interest in LucidAct Health and Gordy Health.

A. Michael Lincoff has received Esperion research funding for this trial; received grants from Eli Lilly, AbbVie, CSL, AstraZeneca, and Novartis; and received personal fees from Novo Nordisk, Glaxo, Akebia, Endologix, Fibrogen, Provention, and Becton Dickson.

Stephen J. Nicholls received research support from AstraZeneca, Amgen, Anthera, CSL Behring, Cerenis, Eli Lilly, Esperion, Resverlogix, Novartis, InfraReDx, and Sanofi‐Regeneron and is a consultant for Amgen, Akcea, AstraZeneca, Boehringer Ingelheim, CSL Behring, Eli Lilly, Esperion, Kowa, Merck, Takeda, Pfizer, Sanofi‐Regeneron, Vaxxinity, CSL Sequiris, and Novo Nordisk.

LeAnne Bloedon, Na Li, Paula Robinson, and Stephanie Kelly are employees of Esperion Therapeutics Inc. and own shares of Esperion stock.

William J. Sasiela is a former employee and current consultant for Esperion Therapeutics Inc. and owns shares of Esperion stock.

Steven E. Nissen reports that the Cleveland Clinic Center for Clinical Research has received funding to perform clinical trials from Abbvie, AstraZeneca, Amgen, Bristol Myers, Squibb, Eli Lilly, Esperion, Medtronic, MyoKardia, New Amsterdam Pharmaceuticals, Novartis, Pfizer, and Silence Therapeutics. Dr. Nissen is involved in these clinical trials but receives no personal remuneration for his participation. Dr. Nissen consults for these pharmaceutical companies but does not accept compensation. The other authors declare no conflicts of interest.

## Data Availability

The data that support the findings of this study are available from the corresponding author upon reasonable request.
